# Targeting Heme Oxygenase-1 in the Arterial Response to Injury and Disease

**DOI:** 10.3390/antiox9090829

**Published:** 2020-09-04

**Authors:** William Durante

**Affiliations:** Department of Medical Pharmacology and Physiology, University of Missouri, Columbia, MO 65212, USA; durantew@health.missouri.edu; Tel.: +1573-882-3886; Fax: 1573-884-4276

**Keywords:** heme oxygenase-1, carbon monoxide, bilirubin, vascular smooth muscle cells, arterial remodeling, vascular disease

## Abstract

Heme oxygenase-1 (HO-1) catalyzes the degradation of heme into carbon monoxide (CO), iron, and biliverdin, which is rapidly metabolized to bilirubin. The activation of vascular smooth muscle cells (SMCs) plays a critical role in mediating the aberrant arterial response to injury and a number of vascular diseases. Pharmacological induction or gene transfer of HO-1 improves arterial remodeling in animal models of post-angioplasty restenosis, vascular access failure, atherosclerosis, transplant arteriosclerosis, vein grafting, and pulmonary arterial hypertension, whereas genetic loss of HO-1 exacerbates the remodeling response. The vasoprotection evoked by HO-1 is largely ascribed to the generation of CO and/or the bile pigments, biliverdin and bilirubin, which exert potent antioxidant and anti-inflammatory effects. In addition, these molecules inhibit vascular SMC proliferation, migration, apoptosis, and phenotypic switching. Several therapeutic strategies are currently being pursued that may allow for the targeting of HO-1 in arterial remodeling in various pathologies, including the use of gene delivery approaches, the development of novel inducers of the enzyme, and the administration of unique formulations of CO and bilirubin.

## 1. Introduction

Arteries transport blood from the heart to all other tissues and organs. They consist of multiple cell types and structural proteins arranged in three concentric layers: the tunica intima, the tunica media, and the tunica adventitia [1]. The tunica intima forms the innermost layer of the vessel, and it consists of a single layer of endothelial cells that serves as a barrier between the blood-carrying lumen and vessel wall. The tunica media is situated between the internal and external elastic laminas and is comprised almost exclusively of circumferentially oriented vascular smooth muscle cells (SMCs). The tunica adventitia is the outermost layer that contains fibroblasts, progenitor cells, and extracellular matrix that maintains the structural integrity of the blood vessel. Arteries are constantly exposed to hemodynamic forces and biochemical stimuli that triggers functional and adaptive responses in one or all three layers of the vessel wall.

Vascular remodeling is a salient feature in aging, but it also occurs in response to injury and disease [2]. Multiple mechanisms are involved in promoting pathological remodeling of the vasculature, including fibrosis, hyperplasia and hypertrophy of the media and intima, alterations in vascular collagen and elastin content, endothelial dysfunction, inflammation, and arterial calcification. Vascular SMCs play a pivotal role in arterial remodeling. They are the most abundant cell type in arteries and are essential for preserving vessel structure and function. Despite being highly specialized, SMCs retain remarkable plasticity. Under physiologic conditions, vascular SMCs express a distinct collection of proteins that contribute to a contractile phenotype that regulates blood pressure and flow throughout the vascular system. However, following arterial injury or in response to pathologic stimuli, SMCs undergo phenotypic switching where they lose their contractility markers and differentiate to a synthetic phenotype [3]. These synthetic cells display high rates of proliferation and synthesize matrix metalloproteinases that promotes SMC migration from the media to the intima by separating these cells from the basement membrane and extracellular matrix. This leads to the formation of a neointima that impairs blood flow. Synthetic SMCs also secrete collagen and other extracellular proteins, which further promotes medial and intimal expansion. Under certain conditions, SMCs can also assume an osteogenic phenotype resulting in the calcification and stiffening of arteries [4]. Moreover, in vivo lineage tracing and fate mapping systems reveal that SMCs can undergo phenotypic transitions into many other cell types, including macrophage-like cells, foam cells, mesenchymal-like stem cells, myofibroblasts, and beige-like adipocytes, suggesting the contribution of SMCs to a host of vascular pathologies [5]. 

Aberrant arterial remodeling contributes to a number of vascular diseases, including restenosis following percutaneous coronary interventions, atherosclerosis, post-transplant vasculopathy, vein graft occlusion, pulmonary arterial hypertension, and vascular access failure [6,7,8,9]. Given the important contribution of vascular SMCs to arterial remodeling, the targeting of these cells offers a possible strategy for therapeutic intervention. Accumulating evidence over the past three decades has identified the enzyme heme oxygenase-1 (HO-1) as a critical regulator of cardiovascular health and disease [10,11,12,13,14,15,16,17,18]. Diverse mechanisms appear to mediate the protective actions of HO-1 in the circulation, including anti-inflammatory and antioxidant effects; anti-thrombotic actions; inhibition of vasomotor tone; and its ability to modulate the growth, function, and survival of vascular cells. This review will discuss the effects of HO-1 on arterial remodeling in specific pathologic states, focusing on its actions on vascular SMCs. In addition, it will highlight potential therapeutic modalities in targeting HO-1 and its products in the treatment of occlusive vascular disease.

## 2. Regulation of HO-1 Activity and Expression

**Figure 1:**
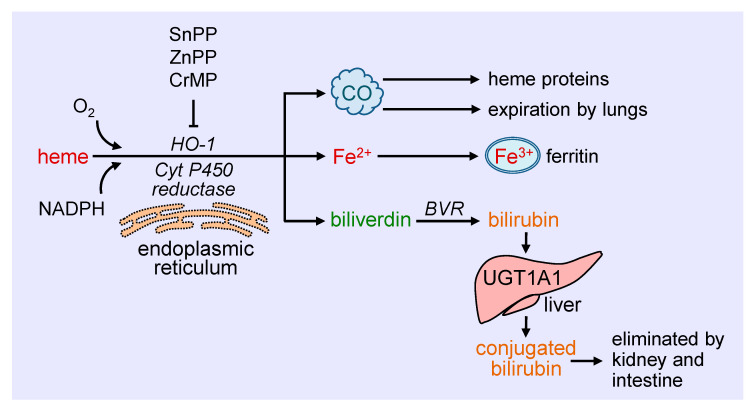
Schematic representation of the metabolism of heme. Heme is metabolized at the endoplasmic reticulum via the concerted action of heme oxygenase-1 (HO-1) and cytochrome P450 (Cyt P450) reductase to carbon monoxide (CO), ferrous iron (Fe^2+^), and biliverdin. The generated CO interacts with intracellular heme proteins or diffuses to the circulation and is transported to the lungs and cleared by exhalation. Fe^2+^ is rapidly sequestered by ferritin and stored in ferric (Fe^3+^) form, while biliverdin is converted to bilirubin by biliverdin reductase (BVR). Ferritin is found in the cytosol of most tissues, but a small fraction is secreted into the blood where it functions as an iron carrier. Bilirubin is removed from the circulation by the liver and metabolized by uridine 5′-diphospho-glucuronlytransferase 1A1 (UGT1A1) to water-soluble conjugated bilirubin for elimination by the intestine and kidney. The oxidative degradation of heme is prevented by various metalloporphyrins, including tin protoporphyrin (SnPP), zinc protoporphyrin (ZnPP), and chromium mesoporphyrin (CrMP).

Heme oxygenase-1 (HO-1) catalyzes the degradation of heme to carbon monoxide (CO), ferrous iron, and biliverdin (Figure 1). This reaction requires molecular oxygen and electrons from nicotinamide adenine dinucleotide phosphate (NADPH) that are provided by cytochrome P450 reductase [19]. The oxidation of heme is inhibited in a competitive manner by a number of metalloporphyrins, including zinc protoporphyrin (ZnPP), tin protoporphyrin (SnPP), and chromium mesoporphyrin [20]. Biliverdin generated from this reaction is subsequently metabolized to bilirubin by biliverdin reductase, while ferrous iron is rapidly sequestered by ferritin and stored in ferric form [21,22]. Ferritin is detected in the cytosol of most tissues, but a small fraction is secreted into the blood where it functions as an iron carrier. Bilirubin is removed from the circulation by the liver and metabolized by uridine 5′-diphospho-glucuronlytransferase 1A1 (UGT1A1) to water-soluble conjugated bilirubin for elimination by the intestine and kidney. The generated CO readily interacts with intracellular heme proteins or diffuses into the circulation and is transported to the lungs and cleared by exhalation. HO-1 is a ubiquitously expressed enzyme that is anchored via its carboxyl terminus to the endoplasmic reticulum where it co-localizes and interacts with cytochrome P450 reductase for optimal enzymatic activity [23]. However, HO-1 has also been localized to other intracellular compartments, including the nucleus, plasma membrane caveolae, and mitochondria [24,25,26,27]. The nuclear localization of HO-1 is associated with the truncation of the carboxyl terminus of the protein and loss of enzymatic activity, and likely plays a role in gene regulation. In addition, HO-1 is targeted to caveolae via its direct interaction with caveolin-1 and caveolin-2 [25,26]. The association with caveolin-1 represses HO-1 activity by interfering with the binding of heme to the enzyme [28]. In addition, minor amounts of HO-1 are observed in the inner mitochondrial membrane of the liver where it modulates mitochondrial heme content and metabolism [24]. The molecular mechanisms involved in the intracellular trafficking of HO-1 are not well defined, and the significance of the different subcellular locations of the enzyme necessitates further study. 

HO-1 is a highly inducible enzyme and is upregulated by a large number of biochemical and biophysical stimuli, including its substrate heme [29]. The control of HO-1 expression occurs largely at the transcriptional level and is mediated by a myriad of transcription factors with nuclear factor E2-related factor-2 (Nrf2) serving as a primary inducer while BTB domain and CNC homolog 1 (Bach1) acts as a major repressor [30]. In humans, a GT-repeat polymorphism in the HO-1 promoter modulates the degree of transcription with long polymorphisms associated with lower inducibility of the gene and increased risk of cardiovascular disease [31]. In addition, two single nucleotide polymorphisms (T(-143)A and G(-1135)A) in the proximal HO-1 promoter have been identified to modulate basal promoter activity, but their clinical significance remains to be established [32,33]. 

## 3. Effect of HO-1 on Vascular SMC Function

HO-1 is a critical regulator of vascular SMC function. Overexpression of HO-1 blocks SMC proliferation and DNA synthesis both in vitro and in vivo [34]. Furthermore, SMCs isolated from HO-1-deficient mice exhibit greater rates of cell growth relative to cells derived from wild-type animals. Moreover, induction of HO-1 by many endogenous factors and pharmaceutical compounds blocks the proliferation of vascular SMCs [10,11,12,13,14,15,16,17,18]. The inhibition of SMC growth by HO-1 is associated with an increase in p21 expression and the arrest of SMCs in the G_0_/G_1_ phase of the cell cycle, whereas inhibition of HO-1 activity promotes cell cycle progression [34,35]. The anti-proliferative effect of HO-1 is dependent on soluble guanylate cyclase as inhibition of this enzyme or its downstream effector, protein kinase G, restores SMC growth [34]. Gene transfer of HO-1 also blocks platelet-derived growth factor (PDGF)-induced migration of vascular SMCs via the assembly of an inactive vascular endothelial growth factor receptor-2/PDGF receptor β heterodimer that retards effective PDGF signaling [36].

Both CO and biliverdin/bilirubin contribute to the anti-proliferative and anti-migratory action of HO-1. In an early study, we showed that the CO-scavenger, hemoglobin, augments mitogenesis in vascular SMCs following the induction of HO-1 [37]. In addition, direct exposure of cultured SMCs to CO gas blocks cell proliferation, cell cycle progression, and DNA synthesis [37,38]. The arrest of SMCs in the G_0_/G_1_ phase of the cell cycle is associated with a rise in p21 expression and a decline in cyclin A levels, cyclin-dependent kinase-2 activity, and phosphorylation of retinoblastoma protein. Multiple mechanisms are involved in the anti-proliferative action of the gas, including activation of soluble guanylate cyclase and p38 mitogen-activated protein kinase (MAPK), inhibition of T-type calcium channels, and the expression of caveolin-1 [39,40,41,42]. Treatment of vascular SMCs with biliverdin or bilirubin also blocks their replication and movement through the cell cycle [43,44,45]. The inhibition of SMC growth by these pigments appears to involve the suppression of the rapidly accelerated fibrosarcoma (Raf)/extracellular-signal-regulated kinase (ERK)/MAPK signaling pathway, induction of p53 expression, hypophosphorylation of retinoblastoma protein, and the proteolysis of the transcription factor Yin Yang 1 (YY1), without any effect on ATP production or mitochondrial function [45,46,47,48]. Interestingly, culture conditions dictate the response of SMCs to bilirubin. While a potent inhibition of SMC replication is noted under serum-replete conditions, apoptosis is observed in cells grown in serum-free or serum-restricted conditions [47,48]. Finally, CO attenuates the migration of cultured vascular SMCs by blocking the activity of NADPH oxidase 1 [49,50]. Similarly, bilirubin blocks the migration of vascular SMCs in a concentration-dependent manner [45].

HO-1 is also an important modulator of vascular SMC viability. Our laboratory was the first to show that the induction of HO-1 in SMCs functions in an adaptive manner to preserve cell survival. Treatment of SMCs with various inflammatory cytokines stimulates apoptosis, as demonstrated by DNA laddering, annexin V binding, and caspase-3 activation. This apoptotic response is potentiated following HO-1 inhibition by ZnPP or by the scavenging of CO with hemoglobin [51]. Furthermore, exogenous administration of CO gas inhibits cytokine-mediated apoptosis in a manner that is partially dependent on the activation of soluble guanylate cyclase and is associated with the inhibition of mitochondrial cytochrome c release and with the suppression of p53 expression [51,52]. The induction of HO-1-derived CO, but not biliverdin, bilirubin or iron, also acts in autocrine fashion to inhibit SMC apoptosis in response to endoplasmic reticulum stress by suppressing the expression of the pro-apoptotic protein, GADD153 [53]. In contrast, bilirubin mediates the anti-apoptotic effect of hypoxia in pulmonary arterial SMCs via the activation of the ERK1/2 pathway [54]. In addition, increases in HO-1 expression by nitrosative stress counteract nitric oxide-induced apoptosis of vascular SMCs [55,56]. The protection afforded by HO-1 involves an increase in mitochondrial membrane potential and Bcl-2 expression along with a decrease in nitric oxide-mediated mitochondrial cytochrome c release, and Bax and Apaf-1 expression. Overexpression of HO-1 also protects SMCs against oxidative injury, while the upregulation of HO-1 blocks apoptosis and the production of reactive oxygen species (ROS) by SMCs [57,58,59,60,61,62]. Collectively, these studies establish HO-1 as an essential regulator of vascular SMC proliferation, migration, and survival. 

## 4. Role of HO-1 in the Arterial Response to Mechanical Injury

**Figure 2:**
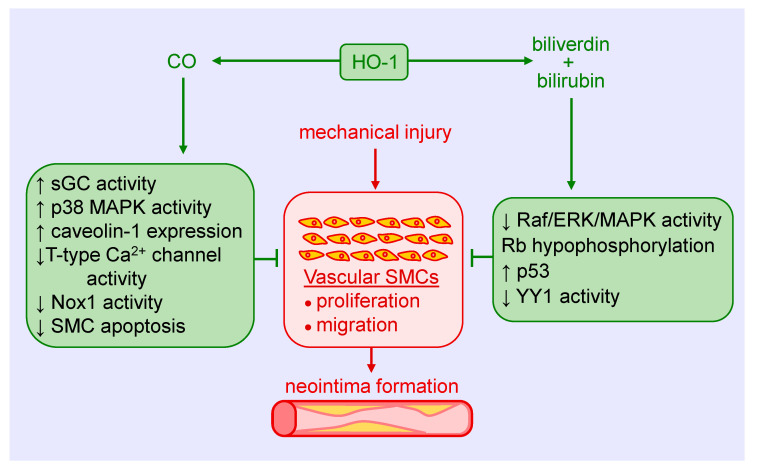
Heme oxygenase-1 (HO-1) inhibits vascular smooth muscle cell (SMC) proliferation and migration, and neointima formation following mechanical injury of arteries via the generation of carbon monoxide (CO), biliverdin, and bilirubin. CO blocks SMC proliferation and migration by stimulating soluble guanylate cyclase (sGC) activity, p38 mitogen-activated protein kinase (MAPK) activity, and caveolin-1 expression, and by reducing T-type calcium (Ca^2+^) channel activity, nicotinamide adenine dinucleotide phosphate oxidase 1 (Nox1) activity, and SMC apoptosis. Biliverdin and bilirubin attenuate SMC proliferation and migration by restraining rapidly accelerated fibrosarcoma (Raf)/extracellular-signal-regulated kinase (ERK)/MAPK activity, retinoblastoma protein (Rb) phosphorylation, and the expression of the transcription factor Yin Yang 1 (YY1), and by increasing p53 expression.

Considerable evidence indicates that HO-1 mitigates intimal thickening in experimental models of angioplasty and stenting (Figure 2). Early work found that the induction of HO-1 by hemin inhibits neointima formation after balloon injury of rat carotid arteries, while blockade of HO-1 activity by metalloporphyrins stimulates intimal thickening [63,64,65]. The induction of HO-1 by hemin also prevents in-stent arterial stenosis in rat and rabbit models [66]. In addition to hemin, a plethora of compounds has been reported to block intimal hyperplasia following arterial injury via the upregulation of HO-1, including probucol, dimethylfumarate, and butylated hydroxyanisole, among others [13,67,68,69]. Consistent with these findings, adenovirus-mediated gene transfer of HO-1 subsequent to arterial injury ameliorates lesion formation in rat carotid and porcine femoral arteries [34,70]. Alternatively, deletion of the HO-1 gene augments intimal hyperplasia following wire injury of mice femoral arteries [34]. Intriguingly, HO-1 may also limit restenosis in humans, as patients with the short (GT)n repeat alleles in the promoter that exhibit higher inducibility of the gene have a reduced risk of restenosis after peripheral percutaneous transluminal angioplasty; however, it is unclear whether this protection occurs following coronary stenting [71,72,73,74,75].

Several studies support the role of CO in reducing neointima formation. Inhalation of CO for two days prior to balloon injury reduces intimal hyperplasia in rat carotid arteries [38]. Similarly, we found that infusion of a saturated solution of CO immediately after balloon injury suppresses intimal hyperplasia of damaged blood vessels [76]. Delayed inhalation of CO starting two weeks after balloon injury also reduces established neointimal lesion size [77]. Mechanistically, the decrease in intimal thickening by CO is dependent on the activation of soluble guanylate cyclase and relies on the p38 MAPK-mediated increase in caveolin-1 expression in neointimal lesions [42]. Interestingly, intimal hyperplasia following carotid artery balloon injury is greatly diminished in Gunn rats, a congenital model of hyperbilirubinemia resulting from the loss of UGT1A1 activity [43]. In addition, systemic administration of biliverdin prior to balloon injury or short local exposure to the pigment before and after injury reduces neointima formation [43]. Subsequently, we discovered that local application of a bilirubin-containing pluronic gel to the adventitia of injured carotid arteries led to a marked decrease in neointima formation [45]. More recently, the coating of everolimus (rapamycin)-containing stents with bilirubin was shown to reduce the inflammatory response following their implantation into coronary arteries of the pig, but the area of restenosis was not significantly improved [78]. Failure of exogenous administration of bilirubin to restrict neointima formation may reflect the ability of rapamycin to stimulate HO-1 gene expression and sufficiently raise intracellular levels of bilirubin in SMCs so that additional increases in bilirubin evoke no further decrease in cell growth [79].

Clinical and experimental studies also implicate HO-1 in maintaining the functionality of the arteriovenous fistula (AVF). The native AVF is the preferred form for dialysis access in patients with end-stage renal disease, but it suffers from frequent failure due to neointimal hyperplasia at the juxta-anastomotic site of the AVF [80]. Interestingly, the long (GT)n repeat alleles of the HO-1 promoter are associated with a higher frequency of access failure and poorer patency of AVFs [81]. We also found that far-infrared radiation induces the expression of HO-1 in vascular cells, and that application of far-infrared therapy improves access blood flow, patency, and inflammation of AVFs in hemodialysis patients [82,83]. However, the effectiveness of far-infrared therapy in preserving the function of AVFs is lessened in hemodialysis patients possessing the long (GT)n repeat polymorphism, suggesting that impaired induction of HO-1 contributes to AVF failure [84]. In support of this notion, robust induction of HO-1 in SMCs of the vein is observed in murine AVF models [85,86]. Furthermore, genetic deficiency of HO-1 impairs the functionality and structure of the AVF. HO-1- null mice exhibit a much lower rate of patency, and this is associated with exuberant venous neointimal hyperplasia, as shown by increases in venous wall thickening and decreases in the luminal area/venous wall cross-section relative to wild-type animals [85]. Moreover, adeno-associated viral gene delivery of HO-1 to the venous wall improves AVF blood flow and decreases venous wall thickness of the AVF in a murine model of chronic kidney disease [87]. The beneficial effects of HO-1 are likely mediated by the generation of CO since exogenous administration of this gas also improves AVF function in mice [86]. 

## 5. Role of HO-1 in Atherosclerosis and Transplant Arteriosclerosis

Atherosclerosis is the most common cause of cardiovascular disease that accounts for nearly one-third of global mortality [88]. Although originally considered a lipid storage disorder, it is a chronic inflammatory disease characterized by the accumulation and oxidation of lipoproteins, inflammatory cells and vascular SMCs within the intima of arteries, endothelial cell dysfunction, and the secretion of inflammatory cytokines and chemokines [89]. Infiltrating monocytes within the artery differentiate into macrophages, engulf lipoproteins becoming lipid-laden foam cells that eventual die to form the necrotic core of the atherosclerotic plaque. A fibrous cap consisting of vascular SMCs and their secreted extracellular matrix proteins provide a protective barrier between the circulating blood and the pro-thrombotic material in the plaque. Rupture of the plaque can result in thrombotic occlusion of the artery, leading to myocardial infarction, stroke, and end-organ failure. 

**Figure 3:**
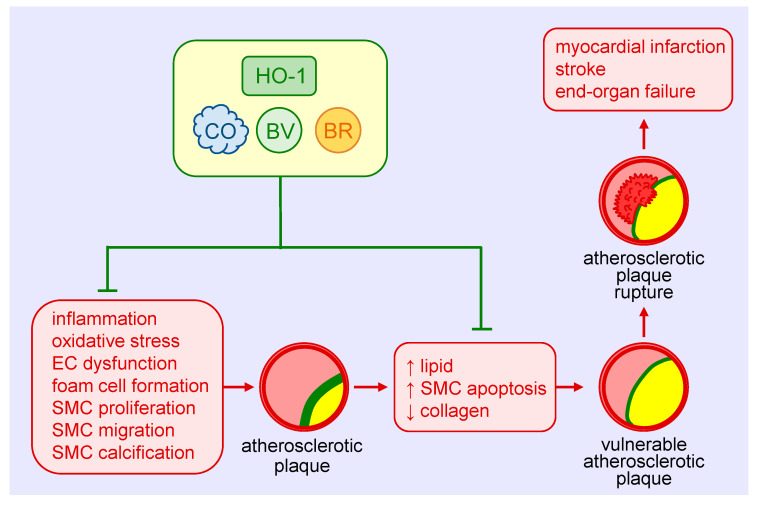
Heme oxygenase-1 (HO-1) inhibits the development and progression of atherosclerosis. HO-1-derived carbon monoxide (CO) and the bile pigments, biliverdin (BV) and bilirubin (BR), avert atherosclerotic plaque formation by blocking vascular inflammation, oxidative stress, endothelial cell (EC) dysfunction, foam cell formation, and vascular smooth muscle cell (SMC) proliferation, migration, and calcification. HO-1 and its products also prevent the development of vulnerable atherosclerotic plaques and plaque rupture as well as thrombosis by limiting lipid accumulation and SMC apoptosis and elevating collagen deposition within arterial lesions. Arterial cross-sections depict the atherosclerotic plaque in yellow, the fibrous cap in green, and thrombus formation in red.

The protective role of HO-1 in atherosclerosis is well-appreciated (Figure 3) and has been extensively reviewed [11,12,13,90]. HO-1 is expressed in various cell types, including SMCs, within atherosclerotic lesions in both humans and animals. Genetic studies suggest that the expression of HO-1 in humans may be of clinical significance. Polymorphisms known to reduce HO-1 promoter activity, including the long (GT)n repeat allele and the A(-143)T single nucleotide polymorphism, are associated with increased atherosclerosis in some patients with coronary artery disease [32,73,91,92,93,94]. Significantly, the presence of fatty streaks and fibrous plaques are present in the aorta of a young boy with HO-1 deficiency [95]. Convincing evidence also supports a protective role of HO-1 against the development of atherosclerosis in various animal models [11,12,13,90]. Pharmacological studies demonstrate that the induction of HO-1 reduces plaque formation, whereas inhibition of HO-1 activity augments plaque size. Similarly, genetic approaches that elevate HO-1 expression retard lesion formation, while loss of HO-1 expression increases atherosclerotic lesions. Multiple mechanisms mediate the beneficial effect of HO-1 in atherosclerosis, including its anti-inflammatory and anti-oxidant effects and its ability to improve endothelial dysfunction and regulate key cellular processes such as proliferation, migration, survival, and differentiation [12]. With respect to SMCs, the ability of HO-1 to block their proliferation and migration limits the intimal accumulation of SMCs in atherosclerotic lesions. Surprisingly, SMCs contribute to the majority of foam cells in human and animal atheromas [96,97]. Whether HO-1 also limits plaque formation by inhibiting the differentiation of SMCs to foam cells remains an open question. However, some evidence suggests that HO-1 may interfere with the differentiation of SMCs to osteoblasts. Overexpression of HO-1 inhibits the maturation and mineralization of osteoblasts, and upregulation of the HO-1-ferritin system prevents osteoblastic differentiation of vascular SMCs via the generation of biliverdin and, to a greater extent, through the ferroxidase activity of ferritin [98,99]. Thus, HO-1 may mitigate the calcification and stiffening of blood vessels in atherosclerosis. The products of HO-1 that mediate the anti-atherosclerotic actions of the enzyme are not fully known. However, a recent report found that bilirubin administration prevents atherosclerotic plaque formation in hyperlipidemic mice by inhibiting the expression of endothelial intercellular adhesion molecule 1 and vascular cell adhesion molecule 1 [100]. Arterial lesions in animals treated with bilirubin exhibit reduced lipid and collagen deposition, fewer inflammatory cells and SMCs, and diminished levels of ROS. The potential role of CO in atherogenesis requires additional investigation.

Aside from inhibiting the development of atherosclerosis, HO-1 may also prevent the progression of the disease by promoting plaque stability. In patients with carotid artery disease, HO-1 expression is increased in lesions with a vulnerable plaque phenotype that is characterized by elevated levels of lipids and diminished amounts of SMCs and collagen [101]. A rise in HO-1 expression is also detected in the vulnerable plaque of mice. Moreover, pharmacological induction of HO-1 or adenoviral-mediated overexpression of HO-1 reduces the lipid concentration and size of the necrotic core, and increases SMC content and fibrous cap thickness in mice, while inhibition of HO-1 activity reverses the properties of stable plaques. Induction of HO-1 also increases fibrous cap thickness and deters plaque rupture in a rabbit model of atherosclerosis [102]. Since the apoptosis of vascular SMCs is a key event in the development of vulnerable plaques [103], the ability of HO-1 to inhibit SMC death may underlie its ability to stabilize atherosclerotic lesions.

Arteriosclerosis of arteries is the main constraint to long-term survival in patients with solid organ transplantation. Transplant arteriosclerosis is characterized by diffuse, concentric narrowing of the artery leading to impaired blood flow to the allograft and eventual organ failure [104]. It is initiated by monocyte infiltration into the vessel wall which triggers a cascade of events leading to the formation of a neointima that is composed primarily of vascular SMCs. Substantial evidence indicates a key role for HO-1 in alleviating transplant arteriosclerosis. HO-1 gene transfer inhibits intimal thickening following aortic transplantation in rats and this is associated with a decrease in the number of SMCs in the lesion [105,106]. In addition, interleukin-10-mediated decreases in neointimal proliferation and inflammation in rat aortic grafts are dependent on HO-1 activity [107]. The protection afforded by HO-1 is mimicked by CO, as administration of methylene chloride, a compound that generates CO following its metabolism by the liver, or inhalation of CO impairs leukocyte infiltration and neointima formation in the transplanted aorta [38,108]. 

HO-1 also promotes the functionality of venous grafts. Resection of a segment of jugular vein into a defect created in an autologous carotid artery of the mouse results in the formation of a neointima [109]. The lesion is much larger in HO-1-knockout mice compared to wild-type animals 10 days after surgery; however, 14 days post-surgery, the neointima of HO-1-null animals is composed primarily of acellular material, indicative of substantial SMC death. These findings suggest a critical role for HO-1 in limiting SMC growth and death in vein grafts. Interestingly, ex vivo delivery of CO or biliverdin to veins prior to implantation into the abdominal aorta of syngeneic rats ameliorates intimal lesion formation, suggesting that both molecules contribute to the salutary effect of HO-1 in arterialized vein grafts [44,110]. Notably, inhalation of CO confers protection against intimal proliferation of SMCs when given perioperatively in a clinically relevant porcine model of arteriovenous grafts [111]. The ability of HO-1 and its products to defend against transplant arteriosclerosis is observed in other tissues, including the heart [112,113,114].

## 6. Role of HO-1 in Pulmonary Arterial Hypertension

Pulmonary arterial hypertension (PAH) is a progressive and severe disease that is largely driven by the hyperproliferation of vascular cells and excessive deposition of extracellular matrix that culminates in the formation of lumen-obliterative lesions in small pulmonary arteries [115,116]. This abnormal remodeling response and the accompanying vasoconstriction elevate pulmonary arterial pressure, leading to right ventricular failure and ultimately death. Multiple factors are linked to the development of PAH, including various inimical stimuli such as hypoxia, infections, toxins, as well as congenital heart disease and a number of heritable genetic mutations. While approved pulmonary vasodilator therapies provide symptomatic relief and improve survival in PAH, there remains a critical need for novel therapies that target the molecular origins of the disease. 

**Figure 4:**
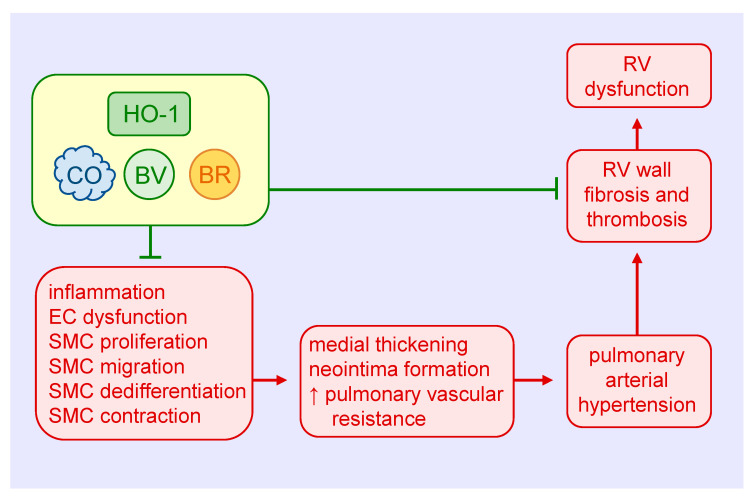
Heme oxygenase-1 (HO-1) inhibits pulmonary arterial remodeling, pulmonary vascular resistance, and right ventricular (RV) dysfunction in pulmonary arterial hypertension. HO-1-derived carbon monoxide (CO) and the bile pigments, biliverdin (BV) and bilirubin (BR), block medial and intimal thickening and pulmonary vascular resistance by attenuating pulmonary inflammation, endothelial cell (EC) dysfunction, and vascular smooth muscle cell (SMC) proliferation, migration, dedifferentiation, and contraction. HO-1 and its products also prevent the dysfunction of the RV in pulmonary arterial hypertension by inhibiting ventricular wall fibrosis and thrombosis.

Ample evidence supports a protective role of HO-1 in the development and progression of PAH (Figure 4). Hypoxia increases the expression of HO-1 mRNA and protein in cultured pulmonary artery SMCs, and elevates HO-1 levels in the lungs of rats exposed to hypoxia [39,117,118]. Moreover, induction of HO-1 by nickel chloride or hemin prevents the hypoxia-mediated development of pulmonary hypertension and the thickening of pulmonary arterioles [117,119]. Hemin treatment also abrogates the induction of pulmonary hypertension and pulmonary arterial wall thickening in rats injected with monocrotaline [119]. In addition, transgenic mice that overexpress HO-1 in the lungs are protected from the development of pulmonary hypertension and vessel wall hypertrophy induced by hypoxia [120]. In contrast, inhibition of HO-1 activity by ZnPP increases mean pulmonary arterial pressure, the percentage of muscularized pulmonary arteries, and the medial thickness and area of pulmonary arteries in rats subjected to hypoxia [121]. Ultrastructural examination also reveals hyperplasia and hypertrophy of endothelial cells and SMCs, and an elevated fraction of SMCs displays a synthetic phenotype in intra-acinar pulmonary muscularized arteries of hypoxic rats treated with the HO-1 inhibitor. Consistent with this latter finding, ZnPP causes an increase in collagen expression in pulmonary arteries in rats exposed to hypoxia. Furthermore, HO-1 inhibition abolishes interleukin-10-mediated improvements in pulmonary hypertension, vascular cell proliferation, right ventricular hypertrophy, and mortality in rats treated with monocrotaline [122]. Aside from direct effects on vascular SMC growth, migration, dedifferentiation, and contractility, the favorable effects of HO-1 in PAH involve improvements in endothelial cell function and pulmonary inflammation [117,118,119,120].

Both CO and biliverdin exert protective effects in the heart and pulmonary vasculature of mice exposed to chronic hypoxia. Daily injections of biliverdin prevent right ventricular fibrosis and thrombosis in hypoxic HO-1-null mice, but have no effect on pulmonary arterial wall thickness [123]. In contrast, continuous inhalation of CO attenuates hypoxia-mediated right ventricular systolic pressure and arteriolar remodeling in both HO-1-deficient and wild-type mice, but fails to block the fibrosis and thrombosis in the right ventricle. Continuous CO inhalation also lessens hypoxic pulmonary hypertension in rats, potentially via the activation of large-conductance voltage and calcium-activated potassium channels and subsequent reduction in pulmonary arterial tone [124]. Significantly, daily episodic inhalation of CO reverses established pulmonary hypertension and returns pulmonary vascular architecture to near normal in three distinct animal models of PAH [125]. The restorative function of CO is associated with a simultaneous increase in apoptosis and decrease in proliferation of vascular SMCs. Mechanistically, CO targets the endothelium to stimulate NO production by endothelial nitric oxide synthase, which in turn activates the apoptotic cascade in hyperproliferative SMCs. This finding is at odds with work showing that inhalation of CO promotes the regression of carotid artery intimal lesions in rats by blocking SMC proliferation despite decreases in apoptosis [77]. The reason for these variable results is not known, but may reflect the use of different animal species and pathologies and/or disparate effects of CO in conductive vessels versus resistance arteries. Daily injections of CO also prevent the development pulmonary hypertension and partially reverse established pulmonary hypertension in mouse models [126]. The protection by CO is associated with a reduction in distal pulmonary artery muscularization and SMC replication, and with a rise in p21 expression in the lungs. Notably, the ability of CO to prevent pulmonary hypertension is lost in p21-deficient mice. 

Interestingly, several drugs inhibit PAH via the induction of HO-1. In particular, rapamycin induces HO-1 expression in the lung and inhibits the development of monocrotaline-induced pulmonary hypertension in rats [127]. The protection by rapamycin is negated by SnPP and mimicked by the HO-1 inducer cobalt protoporphyrin. In addition, the cholesterol-lowering drug simvastatin defends against the development of pulmonary hypertension and ameliorates established pulmonary hypertension via an HO-1-dependent pathway in rodent models of PAH [128,129]. Lastly, in a rat model of pediatric PAH, erythropoietin treatment reduces vessel wall thickness and the occlusion rate of intra-acinar vessels; however, this effect is abrogated following the blockade of HO-1 activity [130]. Together, these studies identify HO-1 as promising pharmaceutical target in blocking vascular and cardiac remodeling in PAH. 

## 7. Therapeutic Potential of HO-1 and Its Products in Vascular Remodeling 

**Figure 5:**
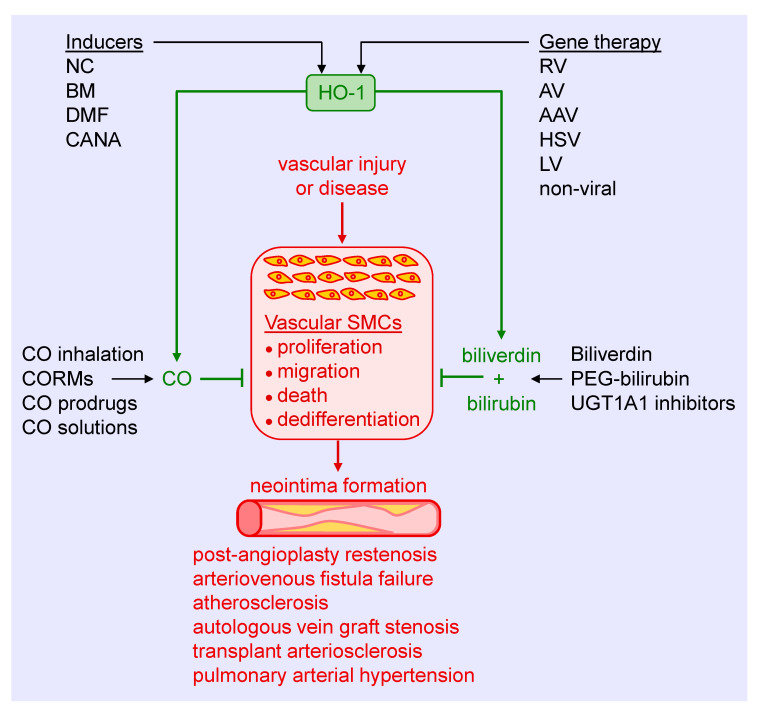
Targeting heme oxygenase-1 (HO-1) and its products in arterial remodeling. Arterial injury or disease triggers the proliferation, migration, death, and dedifferentiation of vascular smooth muscle cells (SMCs), leading to the formation of a neointima. Several strategies may be used to harness the therapeutic potential of HO-1 to block SMC activation and neointima formation in various pathologies. Pharmacological inducers, including nutraceuticals (NC), bardoxolone methyl (BM), dimethylfumurate (DMF), and canagliflozin (CANA), provide a promising approach in stimulating HO-1 expression. Similarly, gene therapy using retroviruses (RV), adenoviruses (AV), adeno-associated viruses (AAV), herpes simplex virus (HSV), lentivirus (LV), or non-viral methods represents an attractive mode of delivering HO-1 to tissues. Alternatively, CO can be administered via gas inhalation or through the use of CO-releasing molecules (CORMs), prodrugs that generate CO, and aqueous solutions of CO. In addition, biliverdin or pegylated bilirubin (PEG-bilirubin) may be directly administered, or pharmacological inhibitors of uridine 5′-diphopho-glucuronlytransferase 1A1 (UGT1A1) alone, or in combination with HO-1 inducers, may be utilized to increase circulating levels of bilirubin.

Substantial experimental evidence has emerged in the past three decades that identifies HO-1 as a crucial regulator of the arterial response to injury and disease. Several strategies can be employed to harness the therapeutic potential of HO-1, including the use of pharmacological inducers, gene therapy, and the exogenous administration of its products CO, biliverdin, and bilirubin (Figure 5). Preclinical studies supports the use of pharmacological inducers of HO-1. Heme and its derivatives are potent inducers of HO-1 that prevent excessive arterial remodeling in various pathological states. These molecules stimulate HO-1 gene expression by binding and inactivating Bach1, thereby facilitating the interaction of Nrf2 with the antioxidant response element of the HO-1 promoter to trigger gene transcription [131]. Moreover, heme is a substrate for HO-1 that may serve to amplify the synthesis of CO and biliverdin since HO-1 activity appears to be substrate-limited in vascular SMCs [132]. Heme-based compounds have been approved by the United States Food and Drug Administration for the treatment of acute porphyria and other red blood cell-related disorders, facilitating their testing in patients. While initial clinical studies demonstrated the ability of hemin or heme arginate infusions to elevate HO-1 levels in peripheral blood mononuclear cells and the heart, respectively, a subsequent investigation found that induction of HO-1 by hemin is short-lived and does not persist for more than one week [133,134,135]. In addition, cobalt protoporphyrin, a widely used heme-based inducer of HO-1, provokes adverse effects such as hepatic, thyroid, and testicular dysfunction [136,137]. Of further concern, heme is a danger-associated molecular pattern (DAMP) molecule that binds to the Toll-like receptor-4 on immune cells to trigger a wave of pro-inflammatory signaling and cytokine release [138]. In fact, heme contributes to the pathogenesis and mortality of severe infections such as malaria and sepsis [139,140]. Given the prominent component of inflammation in cardiovascular disease, the use of heme in this setting needs careful scrutiny. 

Since Nrf2 plays a key role in stimulating HO-1 gene transcription, research has focused on identifying compounds that activate this transcription factor. In this respect, many nutraceuticals have been identified that activate Nrf2 and induce HO-1 expression, including curcumin, resveratrol, cafestol, ginseng, catechins, sulforaphane, lipoic acid, and several flavonoids [141,142]. However, their limited solubility and oral bioavailability have hampered progress in the area. Bardoxolone methyl, a synthetic triterpenoid, is a potent activator of the Nrf2-HO-1 signaling pathway that has yielded encouraging results in some, but not all, patients with diabetic kidney disease [143,144,145]. Dimethylfumurate, approved for use in multiple sclerosis, is another activator of the Nrf2-HO-1 cascade that has shown efficacy against intimal thickening in animals and is under further development [69,146]. More recently, we identified the sodium-glucose co-transporter-2 (SGLT2) inhibitor canagliflozin as a novel inducer of HO-1 in vascular SMCs [147]. The induction of HO-1 by canagliflozin occurs via the activation of Nrf2 through the release of superoxide by the mitochondria. Significantly, the induction of HO-1 by canagliflozin contributes to the anti-proliferative and anti-migratory actions of the drug in SMCs. Notably, these actions are unique for canagliflozin and not seen with other SGLT2 inhibitors. Thus, the ability of canagliflozin to stimulate the expression of HO-1 may contribute to the beneficial cardiovascular actions of this drug in patients with type 2 diabetes mellitus [148]. One concern with using Nrf2 activators as inducers of HO-1 involves their limited selectivity, as Nrf2 triggers the expression of a multitude of genes that may elicit off-target effects.

As previously reviewed [10,11,12,13,14,15,16,17,18,149], many vanguard drugs used to treat cardiovascular disease stimulate the expression of HO-1, including statins, aspirin, nitrates, sildenafil, rapamycin, and paclitaxel. Furthermore, the induction of HO-1 by these drugs is speculated to contribute to their clinical efficacy. However, few studies have examined the ability of pharmacologically relevant concentrations of these drugs to stimulate HO-1 expression in humans. Of note, one study failed to show the induction of HO-1 in healthy subjects treated with therapeutic doses of aspirin, simvastatin, or lipoic acid [150], while another report found that atorvastatin is ineffective in stimulating HO-1 expression in patients with stable angina [151]. These studies emphasize the need for further translational studies in this area. Another challenge associated with the use of pharmacological inducers is the presence of polymorphisms in the promoter that restricts the induction of HO-1. Thus, screening for the HO-1 genotype may be necessary in order to select optimal patient populations for pharmacological intervention. The use of viral or non-viral-mediated gene delivery obviates the need for patient selection. In addition, it has proven to be highly effective in limiting arterial lesions in animal models [34,70,87]. However, concerns related to safety and efficacy have delayed the initiation of HO-1 gene therapy trials. 

The direct administration of the products of the HO-1 reaction provides another therapeutic modality for treating vascular remodeling. The salutary effects of CO have received considerable attention, triggering the development of several delivery platforms. Inhalation of CO has been successfully applied in preclinical studies, but its translation to the clinic remains elusive. Numerous clinical trials have explored the feasibility and efficacy of acute, episodic inhalation of CO in various patient populations with limited success [152]. Failure of the CO inhalation protocols may reflect safety concerns, restricting the dosing of the gas and pharmacokinetic issues that may have led to suboptimal exposure duration. In addition, CO binding to hemoglobin in the blood may have limited diffusion of the gas to tissues beyond the lung. To circumvent these concerns, a novel membrane-based controlled extracorporeal CO releasing system that allows for immediate onset of therapeutic CO levels and continuous monitoring of systemic CO concentrations was designed [153]. In addition, a series of CO-releasing molecules (CORMs) have been constructed that liberates CO in a controlled fashion while minimizing increases in circulating carboxyhemoglobin [154,155]. CORMs possess distinct biophysical properties and release kinetics, and can be engineered to discharge CO in response to specific stimuli, including light, oxidative stress, and pH. More recently, hybrid HO-1 inducing CORMs have been synthesized that contain a CO-releasing moiety coordinated to a known Nrf2 activator [156,157]. These compounds initially release CO, followed by the upregulation of Nrf2-defensive genes, including HO-1 which also stimulates endogenous CO production. The use of organic-based click-and-release prodrugs that utilize a chemical reaction to synthesis CO rather than simple release provides another avenue for CO administration [158]. However, concerns with CORMS and CO-generating prodrugs related to the toxicity of their metal core and possible generation of harmful byproducts, respectively, have impeded their translation to the clinic. Other non-molecular based strategies for CO delivery are also being pursued. One system intended for organ transplantation allows for the controlled release of CO gas from hermetically sealed containers [159]. Furthermore, CO-saturated solutions provides a straightforward vehicle for both acute and chronic administration. We previously reported that brief local delivery of a saturated solution of CO blocks neointima formation in injured rat arteries, while sustained ingestion of an oral formulation of CO decreases vaso-occlusion and inflammation in murine sickle cell disease [76,160]. 

The application of biliverdin and bilirubin provides another option in treating vascular remodeling. Both bile pigments inhibit intimal thickening in preclinical models, driven largely by the inhibition of SMC proliferation and migration [43,44,45]. In addition, clinical studies show that mild elevations in serum bilirubin are sufficient to lower the risk of cardiovascular disease [161,162,163]. In fact, the consumption of bilirubin-rich gallstones for traditional medicinal purposes in Asia is salutary for a host of maladies, providing support for the clinical use of bile pigments [164]. However, there are limitations regarding the deployment of these molecules in humans, including the availability and quality of biliverdin and bilirubin, limited solubility and stability of bilirubin in aqueous solutions, and rapid clearance of bilirubin by the liver. Interestingly, the covalent attachment of polyethylene glycol to bilirubin yields pegylated bilirubin that self-assembles into hydrophilic nanoparticles, obviating some of the solubility issues [165]. Moreover, intravenous administration of pegylated bilirubin is non-toxic and ameliorates inflammation in a murine model of ulcerative colitis [165]. Clearly, additional preclinical studies are needed before this bilirubin formulation is tested in the clinic. Finally, endogenous circulating levels of bilirubin can be increased using pharmacological approaches that prevent the metabolism of bilirubin by UGTA1A. Better still, the simultaneous induction of HO-1 expression and suppression of UGT1A1 activity may optimally raise bilirubin concentrations in the blood. In support of this notion, atazanavir, a joint HO-1 inducer and UGTA1A inhibitor [166,167] raises bilirubin levels and improves vascular function in diabetic patients [168]. Similarly, the drug canagliflozin, which stimulates HO-1 expression and blocks UGTA1A activity [147,169], raises circulating levels of bilirubin in patients with type 2 diabetes, providing further validation for this strategy [170,171].

## 8. Conclusions

Extensive preclinical studies have identified HO-1 as a critical regulator of the arterial response to injury and disease. The vasoprotection afforded by HO-1 occurs via the synthesis of CO, biliverdin and bilirubin that exert potent antioxidant and anti-inflammatory effects. In addition, these molecules inhibit the proliferation, migration, death, and phenotypic switching of vascular SMCs. A number of strategies have been developed and successfully employed to deliver HO-1 and its products in animal models of occlusive vascular disease. Current work is aimed at further refining and translating these positive findings from the laboratory to the clinic.

## References

[1] Mazurek R., Dave J.M., Chandran R.R., Misra A., Sheikh A.Q., Grief D.M. (2017). Vascular cells in blood vessel wall development and disease. Adv. Pharmacol..

[2] Van Varick B.J., Rennenberg R.J.M.W., Reutelingsperger C.P., Kroon A.A., de Leeuw P.W., Schurgers L.J. (2012). Mechanisms of arterial remodeling: Lessons from genetic diseases. Front. Genet..

[3] Owens G.K., Kumar M.S., Wamhoff B.R. (2004). Molecular regulation of vascular smooth muscle cell differentiation in development and disease. Physiol. Rev..

[4] Durham A.L., Speer M.Y., Scatena M., Giachelli C.M., Shanahan C.M. (2018). Role of smooth muscle cells in vascular calcification: Implications in atherosclerosis and arterial stiffness. Cardiovasc. Res..

[5] Liu M., Gomez D. (2019). Smooth muscle cell phenotypic diversity. Arterioscler. Thromb. Vasc. Biol..

[6] Dzau V.J., Brau-Dellaeus R.C., Shedding D.G. (2002). Vascular proliferation and atherosclerosis: New perspectives and therapeutic approaches. Nat. Med..

[7] Jeffrey T.K., Wanstall J.C. (2001). Pulmonary vascular remodeling: A target for therapeutic intervention in pulmonary hypertension. Pharmacol. Ther..

[8] Schiffin E.L. (2012). Vascular remodeling in hypertension: Mechanisms and treatment. Hypertension.

[9] Roostalu U., Wong J.K. (2018). Arterial smooth muscle dynamics in development and repair. Dev. Biol..

[10] Abraham N.G., Kappas A. (2005). Heme oxygenase and the cardiovascular-renal system. Free Rad. Biol. Med..

[11] Durante W. (2010). Targeting heme oxygenase-1 in vascular disease. Curr. Drug. Targets.

[12] Durante W. (2011). Protective role of heme oxygenase-1 against inflammation in atherosclerosis. Front. Biosci..

[13] Ayer A., Zarjou A., Agarwal A., Stocker R. (2016). Heme oxygenase in cardiovascular health and disease. Physiol. Rev..

[14] Drummond G., Baum J., Greenberg M., Lewis D., Abraham N.G. (2019). HO-1 overexpression and underexpression: Clinical implications. Arch. Biochem. Biophys..

[15] Wegiel B., Nemeth Z., Correa-Costa M., Bulmer A.C., Otterbein L.E. (2014). Heme oxygenase-1: A metabolic nike. Antioxid. Redox Signal..

[16] Nitti M., Furfaro A.L., Mann G.E. (2020). Heme oxygenase dependent bilirubin generation in vascular cells: A role in preventing endothelial dysfunction in local tissue environment?. Front. Physiol..

[17] Facchinetti M.M. (2020). Heme oxygenase-1. Antioxid. Redox Signal..

[18] Bellner L., Lebovics N.B., Rubinstein R., Buchen Y.D., Sinatra G., Abraham N.G., McClung J.A., Thompson E.A. (2020). Heme oxygenase-1 upregulation: A novel approach in the treatment of cardiovascular disease. Antioxid. Redox Signal..

[19] Tenhunen R., Marver H.S., Schmid R. (1969). Microsomal heme oxygenase, characterization of the enzyme. J. Biol. Chem..

[20] Kappas A., Drummond G. (1986). Control of heme metabolism with synthetic metalloporphyrins. J. Clin. Investig..

[21] Tenhunen R., Ross M.E., Marver S., Schmid R. (1970). Reduced nicotinamide-adenine dinucleotide phosphate dependent biliverdin reductase: Partial purification and characterization. Biochemistry.

[22] Harrison P.M., Arosio P. (1996). The ferritins: Molecular properties, iron storage function and cellular regulation. Biochim. Biophys. Acta.

[23] Huber W.J., Backes W.L. (2007). Expression and characterization of full-length human heme oxygenase-1: The presence of intact membrane-binding region leads to increased binding affinity for NADPH cytochrome P450 reductase. Biochemistry.

[24] Converso D.P., Taille C., Carreras M.C., Jaitovich A., Poderoso J.J., Boczkowski J. (2006). HO-1 is located in liver mitochondria and modulates mitochondrial heme content and metabolism. FASEB J..

[25] Kim H.P., Wang X., Galbiati F., Ryter S.W., Choi A.M. (2004). Caveolae compartmentalization of heme oxygenase-1 in endothelial cells. FASEB J..

[26] Jung N.-H., Kim H.P., Kim B.-R., Cha S.H., Kim G.A., Ha H., Na Y.E., Cha Y.-N. (2003). Evidence for heme oxygenase-1 association with caveolin-1 and-2 in mouse mesangial cells. IUBMB Life.

[27] Biswasm C., Shah N., Muthu M., La P., Fernando A.P., Sengupta S., Yang G., Dennery P.A. (2014). Nuclear heme oxygenase-1 (HO-1) modulates subcellular distribution and activation of Nrf2, impacting metabolic and antioxidant defenses. J. Biol. Chem..

[28] Taira J., Sugishima M., Kida Y., Oda E., Noguchi M. (2011). Caveolin-1 is a competitive inhibitor of heme oxygenase-1 (HO-1) with heme: Identification of a minimum sequence in caveolin-1 for binding to HO-1. Biochemistry.

[29] Ferrandiz M.L., Devesa I. (2008). Inducers of heme oxygenase-1. Curr. Pharm. Des..

[30] Alam J., Cook J.L. (2003). Transcriptional regulation of the heme oxygenase-1 gene via the stress response pathway. Curr. Pharm. Des..

[31] Exner M., Minar E., Wagner O., Schillinger M. (2004). The role of heme oxygenase-1 promoter polymorphisms in human disease. Free Rad. Biol. Med..

[32] Ono K., Goto Y., Takagi S., Baba S., Tago N., Nonogi H., Iwai N. (2004). A promoter variant of the heme oxygenase-1 gene may reduce the incidence of ischemic heart disease in Japanese. Atherosclerosis.

[33] Zhang G.H., Chen S.M., Wang D.M., Huang X.S. (2010). 413A/T polymorphism in heme oxygenase-1 gene promoter is related to susceptibility of coronary artery disease in patients with dyslipidemia. Chin. J. Arterioscler..

[34] Duckers H.J., Boehm M., True A.L., Yet S.-F., Park J.L., Webb R.C., Lee M.-E., Nabel G.J., Nabel E.G. (2001). Heme oxygenase-1 protects against vascular constriction and proliferation. Nat. Med..

[35] Li Volti G., Wang J., Traganos F., Kappas A., Abraham N.G. (2002). Differential effect of heme oxygenase-1 in endothelial and smooth muscle cell progression. Biochem. Biophys. Res. Commun..

[36] Cheng C., Haasdijk R.A., Tempel D., den Dekker W.K., Chriif I., Blonden L.A.J., va de Kamp E.S.H., de Boer M., Burgisser P.E., Noorderloos A. (2012). PDGF_induced migration of vascular smooth muscle cells is inhibited by heme oxygenase-1 via VEGFR2 upregulation and subsequent assembly of inactive VEGFR2/PDGFRβ heterodimers. Arterioscler. Thromb. Vasc. Biol..

[37] Peyton K.J., Reyna S.V., Chapman G.B., Ensenat D., Liu X., Wang H., Schafer A.I., Durante W. (2002). Heme oxygenase-1-derived carbon monoxide is an autocrine inhibitor of vascular smooth muscle cell growth. Blood.

[38] Otterbein L.E., Zuckerbraun B.S., Haga M., Liu F., Song R., Usheva A., Stachulak C., Bodyak N., Neil Smith R., Czismadia E. (2003). Carbon monoxide suppresses arteriosclerotic lesions associated with chronic graft rejection and with balloon injury. Nat. Med..

[39] Morita T., Perrella M.A., Lee M.A., Kourembanas S. (1995). Smooth muscle cell-derived carbon monoxide is a regulator of vascular smooth muscle cGMP. Proc. Natl. Acad. Sci. USA.

[40] Christodoulides N., Durante W., Kroll M.H., Schafer A.I. (1995). Vascular smooth muscle cell heme oxygenases generate guanylyl cyclase-stimulatory carbon monoxide. Circulation.

[41] Duckles H., Boycott H.E., Al-Owais M.M., Elies J., Johnson E., Dallas M.L., Porter K.E., Giuntini F., Boyle J.P., Scragg J.L. (2015). Heme oxygenase-1 regulates cell proliferation via carbon monoxide-mediated inhibition of T-type Ca2+ channels. Pflugers Arch..

[42] Kim H.P., Wang X., Nakao A., Kim S.I., Murase N., Choi M.E., Ryter S.W., Choi A.M. (2005). Caveolin-1 expression by means of p38β mitogen-activated protein kinase mediates the antiproliferative effect of carbon monoxide. Proc. Natl. Acad. Sci. USA.

[43] Ollinger R., Bilban M., Erat A., Froio A., McDaid J., Tyagi S., Csizmadia E., Graca-Souza A.V., Liloia A., Soares M.P. (2005). Bilirubin: A natural inhibitor of vascular smooth muscle cell proliferation. Circulation.

[44] Nakao A., Murase N., Ho C., Toyokawa H., Billiar T.R., Kanno S. (2005). Biliverdin administration prevents the formation of intimal hyperplasia induced by vascular injury. Circulation.

[45] Peyton K.J., Shebib A.R., Azam M.A., Liu X.M., Tulis D.A., Durante W. (2012). Bilirubin inhibits neointima formation and vascular smooth muscle cell proliferation and migration. Front. Pharmacol..

[46] Stoeckius M., Erat A., Fujikawa T., Hiromura M., Koulova A., Otterbein L.E., Bianchi C., Tobiasch E., Dagon Y., Selke F.W. (2012). Essential roles of Raf/extracellular signal-regulated kinase/mitogen-activated protein kinase pathway, YY1, and Ca2+ influx in growth arrest of human vascular smooth muscle cells by bilirubin. J. Biol. Chem..

[47] Ollinger R., Yamashita K., Bilban M., Erat A., Kogler P., Thomas M., Csizmadia E., Usheva A., Margreiter R., Bach F.H. (2007). Bilirubin and biliverdin treatment of atherosclerotic disease. Cell Cycle.

[48] Liu X.M., Chapman G.B., Wang H., Durante W. (2002). Adenovirus-mediated heme oxygenase-1 gene expression stimulates apoptosis in vascular smooth muscle cells. Circulation.

[49] Rodriguez A.I., Gangopadhyay A., Kelley E.E., Pagano P.J., Zuckerbraun B.S., Bauer P.M. (2010). HO-1 and CO decrease platelet-derived growth factor-induced vascular smooth muscle cell migration via inhibition of Nox1. Arterioscler. Thromb. Vasc. Biol..

[50] Tsai M.H., Lee C.W., Hsu L.F., Li S.Y., Chiang Y.C., Lee M.H., Chen C.H., Liang H.F., How J.M., Chang P.J. (2017). CO-releasing molecules CORM2 attenuates angiotensin II-induced human aortic smooth muscle cell migration through inhibition of ROS/IL-6 generation and matrix metalloproteinase-9 expression. Redox Biol..

[51] Liu X.M., Chapman G.B., Peyton K.J., Schafer A.I., Durante W. (2002). Carbon monoxide inhibits apoptosis in vascular smooth muscle cells. Cardiovasc. Res..

[52] Liu X.M., Chapman G.B., Peyton K.J., Schafer A.I., Durante W. (2003). Antiapoptotic action of carbon monoxide in cultured vascular smooth muscle cells. Exp. Biol. Med. (Maywood).

[53] Liu X.M., Peyton K.J., Ensenat D., Wang H., Schafer A.I., Alam J., Durante W. (2005). Endoplasmic reticulum stress stimulates heme oxygenase-1 gene expression in vascular smooth muscle. Role in cell survival. J. Biol. Chem..

[54] Song S., Wang H., Ma J., Yao L., Xing H., Zhang L., Liao L., Zhu D. (2013). Biliverdin reductase/bilirubin mediates the anti-apoptotic effect of hypoxia in pulmonary arterial smooth muscle cells through ERK1/2 pathway. Exp. Cell Res..

[55] Liu X.M., Peyton K.J., Ensenat D., Wang H., Hannink M., Alam J., Durante W. (2007). Nitric oxide stimulates heme oxygenase-1 gene transcription via the Nrf2/ARE complex to promote vascular smooth muscle cell survival. Cardiovasc. Res..

[56] Kwak H.J., Park K.M., Lee S., Lim H.J., Go S.H., Eom S.M., Park H.Y. (2006). Preconditioning with low concentrations NO attenuates subsequent NO-induced apoptosis in vascular smooth muscle cells via HO-1-dependent mitochondrial death pathway. Toxicol. Appl. Pharmacol..

[57] Zhang M., Zhang B.H., Chen L., An W. (2002). Overexpression of heme oxygenase-1 protects smooth muscle cells against oxidative injury and inhibits cell proliferation. Cell Res..

[58] Brunt K.R., Fenrich K.K., Kiani G., Tse M.Y., Pang S.C., Ward C.A., Melo L.G. (2006). Protection of human vascular smooth muscle cells from H_2_O_2_-induced apoptosis through functional codependence between HO-1 and AKT. Arterioscler. Thromb. Vasc. Biol..

[59] Gabunia K., Ellison S.P., Singh H., Datta P., Kelemen S.E., Rizzo V., Autieri M.V. (2012). Interleukin-19 (IL-19) induces heme oxygenase-1 (HO-1) expression and decreases reactive oxygen species in human vascular smooth muscle cells. J. Biol. Chem..

[60] Schwartz M., Bockmann S., Hinz B. (2018). Up-regulation of heme oxygenase-1 expression and inhibition of disease-associated features of cannabinoid in vascular smooth muscle cells. Oncotarget.

[61] Stulnig G., Frisch M.T., Crnkovic S., Stiegler P., Sereinigg M., Stacher E., Olschewski A., Frank S. (2013). Docahexaenoic acid (DHA)-induced heme oxygenase-1 attenuates cytotoxic effects of DHA in vascular smooth muscle cells. Atherosclerosis.

[62] Yu X., Tao W., Jiang F., Li C., Lin J., Liu C. (2010). Celastrol attenuates hypertension-induced inflammation and oxidative stress in vascular smooth muscle cells via the induction of HO-1. Am. J. Hypertens..

[63] Aizawa T., Ishizaka N., Taguchi J., Kimura S., Kurokawa K., Ohno M. (1999). Balloon injury does not induce heme oxygenase-1 gene expression, but administration of hemin inhibits neointimal formation in balloon injured rat carotid arteries. Biochem. Biophys. Res. Commun..

[64] Togane Y., Toshisuki M., Suematsu M., Ishimura Y., Yamazaki J., Katayama S. (2000). Protective roles of endogenous carbon monoxide in neointimal development elicited by arterial injury. Am. J. Physiol. Heart Circ. Physiol..

[65] Tulis D.A., Durante W., Peyton K.J., Evans A.J., Schafer A.I. (2001). Heme oxygenase-1 attenuates vascular remodeling following balloon injury in rat carotid arteries. Atherosclerosis.

[66] Hyvelin J.M., Maurel B., Uzbekov R., Motterlini R., Lermusiaux P. (2010). Hemin prevents in-stent stenosis in rat and rabbit models by inducing heme oxygenase-1. J. Vasc. Surg..

[67] Deng Y.M., Wu B.J., Witting P.K., Stocker R. (2004). Probucol protects against smooth muscle cell proliferation by upregulating heme oxygenase-1. Circulation.

[68] Liu X.M., Azam M.A., Peyton K.J., Ensenat D., Keswani A., Wang H., Durante W. (2007). Butylated hydroxyanisole stimulates heme oxygenase-1 gene expression and inhibits neointima formation in rat arteries. Cardiovasc. Res..

[69] Oh C.J., Park S., Kim Y.J., Kim H.J., Jeong N.H., Choi Y.K., Go Y., Park K.G., Lee I.K. (2014). Dimethylfumurate attenuates restenosis after acute vascular injury by cell-specific and Nrf2-dependent mechanisms. Redox Biol..

[70] Tulis D.A., Durante W., Liu X.M., Evan A.J., Peyton K.J., Schafer A.I. (2001). Adenovirus-mediated heme oxygenase-1 gene delivery inhibits injury-induced vascular neointima formation. Circulation.

[71] Exner M., Schillinger M., Minar E., Mlekusch W., Schlerka G., Haumer M., Mannhalter C., Wagner O. (2001). Heme oxygenase-1 gene promoter microsatellite polymorphism is associated with restenosis after percutaneous transluminal angioplasty. J. Endovasc. Ther..

[72] Schillinger M., Exner M., Minar E., Mlekusch W., Mullner M., Mannhalter C., Bach F.H., Wagner O. (2004). Heme oxygenase-1 genotype and restenosis after balloon angioplasty: A novel vascular protective factor. J. Am. Coll. Cardiol..

[73] Kaneda H., Ohno J., Tasguchi J., Togo M., Hashimoto H., Ogasawara K., Aizawa T., Ishizaka N., Nagai R. (2002). Heme oxygenase-1 gene promoter polymorphism is associated with coronary artery disease in Japanese patients with coronary risk factors. Arterioscler. Thromb. Vasc. Biol..

[74] Chen Y.-H., Chau L.-Y., Lin M.-W., Chen L.-C., Yo M.-H., Chen J.-W., Lin S.-J. (2004). Heme oxygenase-1 gene promoter microsatellite polymorphism is associated with angiographic restenosis after coronary stenting. Eur. Heart J..

[75] Tiroch K., Koch W., von Beckerath N., Kastrati A., Schomig A. (2007). Heme oxygenase-1 gene promoter polymorphism and restenosis following coronary stenting. Eur. Heart J..

[76] Tulis D.A., Keswani A.N., Peyton K.J., Wang H., Schafer A.I., Durante W. (2005). Local administration of carbon monoxide inhibits neointima formation in balloon-injured rat carotid arteries. Cell. Mol. Biol..

[77] Madigan M., Entabi F., Zuckerbraun B., Loughran P., Tzeng E. (2015). Delayed inhaled carbon monoxide mediates the regression of established neointimal lesions. J. Vasc. Surg..

[78] Bae I.-H., Park D.S., Lee S.-Y., Jang E.-J., Shim J.-W., Lim K.-S., Park J.-K., Kim J.H., Sim D.S., Jeong M.H. (2018). Bilirubin coating attenuates the inflammatory response to everolimus-coated stents. J. Biomed. Mater. Res..

[79] Visner G.A., Lu F., Zhou H., Kazemfar K., Agarwal A. (2003). Rapamycin induces heme oxygenase-1 in human pulmonary vascular cells: Implications in the anti-proliferative response to rapamycin. Circulation.

[80] Durante W., Lin C.-C. (2008). Homing in on arteriovenous fistula survival. Kidney Int..

[81] Lin C.-C., Yang W.-C., Lin S.-J., Chen T.-W., Lee W.-S., Chang C.-F., Lee P.-C., Lee S.-D., Su T.-S., Fann S.S.-Y. (2006). Length polymorphism in heme oxygenase-1 is associated with arteriovenous fistula patency in hemodialysis patients. Kidney Int..

[82] Lin C.-C., Liu X.-M., Peyton K.J., Wang H., Yang W.-C., Lin S.-J., Durante W. (2008). Far infrared therapy inhibits vascular endothelial inflammation via the induction of HO-1. Arterioscler. Thromb. Vasc. Biol..

[83] Lin C.-C., Channg C.F., Lai M.Y., Chen T.-W., Lee P.-C., Yang W.-C. (2007). Far-infrared therapy: A novel treatment to improve access blood flow and unassisted patency of arteriovenous fistula in hemodialysis patients. J. Am. Soc. Nephrol..

[84] Lin C.-C., Chung M.-Y., Yang W.-C., Lin S.-J., Lee P.-C. (2013). Length polymorphisms of heme oxygenase-1 determine the effect of far-infrared therapy on the function of arteriovenous fistula in hemodialysis patients: A novel physicogenomice study. Nephrol. Dial. Transplant..

[85] Juncos J.P., Tracz M.J., Croatt A.J., Grande J.P., Ackerman A.W., Katusic Z.S., Nath K.A. (2008). Genetic deficiency of heme oxygenase-1 impairs functionality and form of an arteriovenous fistula in the mouse. Kidney Int..

[86] Kang L., Yamada S., Hernandez M.C., Croatt A.J., Grande J.P., Juncos J.P., Vercellotti G.M., Hebbel R.P., Katusic Z.S., Terzic A. (2011). Regional and systemic hemodynamic responses following the creation of a murine arteriovenous fistula. Am. J. Physiol. Renal Physiol..

[87] Kang L., Grande J.P., Hillestad M.L., Croatt A.J., Barry M.A., Katusic Z.S., Nath K.A. (2016). A new mouse model of an arteriovenous fistula in chronic kidney disease in the mouse: Beneficial effects of upregulated heme oxygenase-1. Am. J. Physiol. Renal Physiol..

[88] Benjamin E.J., Blaha M.J., Chiuve S.E., Cushman M., Das S.R., Deo R. (2017). Heart disease and stroke statistics-2017 update. Circulation.

[89] Tabas I., Garcia-Cardena G., Owens G.K. (2015). Recent insight into the cellular biology of atherosclerosis. J. Cell. Biol..

[90] Kishimoto Y., Kondo K., Momiyama Y. (2019). The protective role of heme oxygenase-1 in atherosclerotic diseases. Int. J. Mol. Med..

[91] Chen Y.H., Lin S.Y., Lin M.W., Tsai H.L., Kuo S.S., Chen J.W., Chang M.J., Wu T.C., Chen L.C., Ding Y.A. (2002). Microsatellite polymorphism in promoter of heme oxygenase-1 gene is associated with susceptibility to coronary artery disease in type II diabetic patients. Hum. Genet..

[92] Chen Y.-H., Chau L.-Y., Chen J.-W., Lin S.-J. (2008). Serum bilirubin and ferritin levels link heme oxygenase-1 promoter polymorphisms and susceptibility to coronary artery disease in diabetic patients. Diabetes Care.

[93] Endler G., Exner M., Schillinger M., Marculescu R., Sunder-Plassmann R., Raith M., Jordanova N., Wojita J., Mannhalter C., Wagner O.F. (2004). A microsatellite polymorphism in the heme oxygenase-1 gene promoter is associated with increased bilirubin and HDL levels but not with coronary artery disease. Thromb. Haemost..

[94] Qiao H., Sai X., Gai L., Huang G., Chen X., Tu X., Ding Z. (2014). Association between heme oxygenase-1 gene polymorphisms and susceptibility to coronary artery disease: A HuGE review and meta-analysis. Am. J. Epidemiol..

[95] Yachie A., Niida Y., Wada T., Igarishi N., Kaneda H., Toma T., Ohta K., Kasahari Y., Koizumi S. (1999). Oxidative stress causes enhanced endothelial cell injury in human heme oxygenase-1 deficiency. J. Clin. Investig..

[96] Allahverdian S., Chehroudi A.C., McManus B.M., Abraham T., Francis G.A. (2014). Contribution of intimal smooth muscle cells to cholesterol accumulation and macrophage-like cells in human atherosclerosis. Circulation.

[97] Wang Y., Dubland J.A., Allahverdian S., Asonye E., Sahin B., Jaw J.E., Sin D.D., Seidman M.A., Leeper N.J., Francis G.A. (2019). Smooth muscle cells contribute to the majority of foam cells in ApoE (Apolipoprotein E)-deficient mouse atherosclerosis. Arterioscler. Thromb. Vasc. Biol..

[98] Lin T.-Z., Tang C.-H., Hung S.-Y., Liu S.-H., Lin Y.-M., Fu W.-M., Yang R.-S. (2010). Upregulation of heme oxygenase-1 inhibits the maturation and mineralization of osteoblasts. J. Cell Physiol..

[99] Zarjou A., Jeney V., Arosio P., Poli M., Antal-Szalmas P., Agarwal A., Balla G., Jozsef B. (2009). Ferritin prevents calcification and osteoblastic differentiation of vascular smooth muscle cells. J. Am. Soc. Nephrol..

[100] Vogel M.E., Idelman G., Konaniah E.S., Zucker S.D. (2017). Bilirubin prevents atherosclerosis lesion formation in low-density lipoprotein receptor-deficient mice by inhibiting endothelial VCAM-1 and ICAM-1 signaling. J. Am. Heart Assoc..

[101] Cheng C., Noordeloos A.M., Jeney V., Soares M.P., Moll F., Pasterkamp G., Serruys P.W., Duckers H.J. (2009). Heme oxygenase-1 determines atherosclerotic lesion progression into a vulnerable plaque. Circulation.

[102] Li T., Tian H., Zhao Y., An F., Zhang L., Zhang J., Peng J., Zhang Y., Guo Y. (2011). Heme oxygenase-1 inhibits progression and destabilization of vulnerable plaques in a rabbit model of atherosclerosis. Eur. J. Pharmacol..

[103] Clarke M., Bennett M. (2006). The emerging role of vascular smooth muscle cell apoptosis in atherosclerosis and plaque stability. Am. J. Nephrol..

[104] Rahmani M., Cruz R.P., Granville D.J., McManus B.M. (2006). Allograft vasculopathy versus atherosclerosis. Circ. Res..

[105] Bouche D., Chauveau C., Roussel J.C., Mathieu P., Bradeau C., Tesson L., Soulillou J.P., Iyer S., Buelow R., Anegon I. (2002). Inhibition of graft arteriosclerosis development in rat aortas following heme oxygenase-1 gene transfer. Transp. Immunol..

[106] Du D., Chang S., Chen B., Zhou H., Chen Z.K. (2007). Adenovirus-mediated heme oxygenase transfer inhibits graft arteriosclerosis in rat aortic transplants. Transpl. Proc..

[107] Chen S., Kapturczak M.H., Wasserfall C., Glushakova O., Campbell-Thompson M., Deshane J.S., Joseph R., Cruz P.E., Hauswirth W.W., Madsen K.M. (2005). Interleukin-10 attenuates neointimal proliferation and inflammation in aortic allografts by a heme oxygenase-dependent pathway. Proc. Natl. Acad. Sci. USA.

[108] Chauveau C., Bouchet D., Roussel J.-P., Mathieu P., Braudeau C., Renaudin K., Tesson L., Soullilou J.-P., Iyer S., Buelow R. (2002). Gene transfer of heme oxygenase-1 and carbon monoxide delivery inhibit chronic rejection. Am. J. Transpl..

[109] Yet S.F., Layne M.D., Liu X., Chen Y.H., Ith B., Sibinga N.E., Perrella M.A. (2003). Absence of heme oxygenase-1 exacerbates atherosclerosis lesion formation and vascular remodeling. FASEB J..

[110] Nakao A., Huang C.-S., Stolz D.B., Wang Y., Franks J.M., Tochigi N., Billiar T.R., Toyoda Y., Tzeng E., McCurry K.R. (2011). Ex vivo carbon monoxide delivery inhibits intimal hyperplasia in arterialized vein grafts. Cardiovasc. Res..

[111] Ramlawi B., Scott J.R., Feng J., Mieno S., Raman K.G., Gallo D., Csizmadia E., Chin B.Y., Bach F.H., Otterbein L.E. (2007). Inhaled carbon monoxide prevents graft-induced intimal hyperplasia in swine. J. Surg. Res..

[112] Soares M.P., Lin Y., Anrather J., Csizmadia E., Takigami K., Sato K., Grey S.T., Colvin R.B., Choi A.M., Poss K.D. (1998). Expression of heme oxygenase-1 can determine cardiac xenograft survival. Nat. Med..

[113] Hancock W.W., Buelow R., Sayegh M.H., Turka L.A. (1998). Antibody-induced transplant arteriosclerosis is prevented by graft expression of anti-oxidant and anti-apoptotic genes. Nat. Med..

[114] Musameh M.D., Green C.J., Mann B.E., Fuller B.J., Motterlini R. (2007). Improved myocardial function after cold storage with preservation solution supplemented with carbon monoxide-releasing molecule (CORM3). J. Heart Lung Transpl..

[115] Chan Y., Loscalzo J. (2008). Pathogenic mechanisms of pulmonary arterial hypertension. J. Mol. Cell. Cardiol..

[116] Rabinovitch M. (2012). Molecular pathogenesis of pulmonary arterial hypertension. J. Clin. Investig..

[117] Christou H., Morita T., Hsieh C.-M., Koike H., Arkonac B., Perrella M.A., Kourembanas S. (2000). Prevention of hypoxia-induced pulmonary hypertension by enhancement of endogenous heme oxygenase-1 in the rat. Circ. Res..

[118] Zhen G., Zhang Z., Xu Y. (2003). The role of endogenous carbon monoxide in the hypoxic vascular remodeling of rat model of hypoxic pulmonary hypertension. J. Huazhong Univ. Sci. Technolog. Med. Sci..

[119] Shimzu K., Takahashi T., Iwasaki T., Shimzu H., Inoue K., Morimatsu H., Omori E., Matsumi M., Akagi R., Morita K. (2008). Hemin treatment abrogates monocrotaline-induced pulmonary hypertension. Med. Chem..

[120] Minamino T., Christou H., Hsieh C.M., Liu Y., Dhawan V., Abraham N.G., Perrella M.A., Mitsialis S.A., Kourembanas S. (2001). Targeted expression of heme oxygenase-1 prevents the pulmonary inflammatory and vascular responses to hypoxia. Proc. Natl. Acad. Sci. USA.

[121] Gong L., Du J., Shi L., Shi Y., Tang C. (2004). Effects of endogenous carbon monoxide on collagen synthesis in pulmonary artery in rats under hypoxia. Life Sci..

[122] Ito T., Okada T., Miyashita H., Nomoto T., Nonoka-Sarukawa M., Uchibori R., Maeda Y., Urabe M., Mizukami H., Kume A. (2007). Interleukin-10 expression mediated by an adeno-associated virus vector prevents monocrotaline-induced pulmonary arterial hypertension in rats. Circ. Res..

[123] Vitali S.H., Mitsialis S.A., Liang O.D., Liu X., Fernandez-Gonzalez A., Christou H., Wu X., McGowan F.X., Kourembanas S. (2009). Divergent cardiopulmonary actions of heme oxygenase enzymatic products in chronic hypoxia. PLoS ONE.

[124] DuBuis E., Potier M., Wang R., Vandier C. (2005). Continuous inhalation of carbon monoxide attenuates hypoxic pulmonary hypertension development presumably through activation of BKCa channels. Cardiovasc. Res..

[125] Zuckerbraun B.S., Chin B.Y., Wegiel B., Billiar T.R., Czismadia E., Rao J., Shimoda L., Ifedigo E., Kanno S., Otterbein L.E. (2006). Carbon monoxide reverses established pulmonary hypertension. J. Exp. Med..

[126] Abid S., Houssaini A., Mouraret N., Marcos E., Amsellem V., Wan F., Dubois-Rande J.L., Derumeaux G., Boczkowski J., Motterlini R. (2014). P21-dependent protective effects of a carbon monoxide-releasing molecule-3 in pulmonary hypertension. Arterioscler. Thromb. Vasc. Biol..

[127] Zhou H., Liu H., Porvasnik S.L., Terada N., Agarwal A., Chen Y., Visner G.A. (2006). Heme oxygenase-1 mediates the protective effects of rapamycin in monocrotaline-induced pulmonary hypertension. Lab. Investig..

[128] Zhang W.-H., Zhang Y.-J., Liu C.-P., Yu B.-X., Lu W.-X. (2011). Simvastatin protects against development of monocrotaline-induced pulmonary hypertension in rats via a heme oxygenase-1 dependent pathway. Exp. Lung Res..

[129] Hsu H.-H., Ko W.-J., Hsu J.-Y., Chen J.-S., Lee Y.-C., Lai I.-R., Chen C.-F. (2009). Simvastatin ameliorates established pulmonary hypertension through a heme oxygenase-1 dependent pathway in rats. Respir. Res..

[130] Van Loon R.L.E., Bartelds B., Wagener F.A.D.T.G., Affara N., Mohaupt S., Wijnberg H., Pennings S.W.C., Takens J., Berger R.M.F. (2015). Erythropoietin attenuates pulmonary vascular remodeling in experimental pulmonary arterial hypertension through the interplay between endothelial progenitor cells and heme oxygenase. Front. Pediatr..

[131] Sun Y., Rotenberg M.O., Hoshino H., Takaku K., Nakajima O., Muto A., Suzuki H., Tashiro S., Shibahara S., Alam J. (2002). Hemoprotein Bach1 regulates enhancer availability of heme oxygenase-1 gene. EMBO J..

[132] Morimoto Y., Durante W., Lancaster D.G., Klattenhoff J., Tittel F.K. (2001). Real-time measurements of endogenous CO production from vascular cells using an ultrasensitive laser sensor. Am. J. Physiol. Heart Circ. Physiol..

[133] Bharucha A.E., Kulkarni A., Choi K.M., Camilleri M., Lempke M., Brunn G.J., Gibbons S.J., Zinsmeister A.R., Farrugia G. (2010). First-in-human study demonstrating pharmacological activation of heme oxygenase-1 in humans. Clin. Pharmacol. Ther..

[134] Andreas M., Oeser C., Kainz F.M., Shabanian S., Aref T., Bilban M., Messner B., Heidtmann J., Laufer G., Kocher A. (2018). Intravenous heme arginate induces HO-1 (heme oxygenase-1) in the human heart: Randomized, placebo-controlled, safety, and feasibility pharmacokinetic study. Arterioscler. Thromb. Vasc. Biol..

[135] Bharucha A.E., Daley S.L., Low P.A., Gibbons S.J., Choi K.M., Camilleri M., Saw J.J., Farrugia G., Zinsmeister A.R. (2016). Effect of hemin on heme oxygenase-1, gastric emptying, and symptoms in diabetic gastroparesis. Neurogastroenterol. Motil..

[136] Muhoberac B.B., Hanew T., Halter S., Schenker S. (1989). A model of cytochrome-450-centerd hepatic dysfunction in drug metabolism induced by cobalt protoporphyrin administration. Biochem. Pharmacol..

[137] Smith T.J., Drummond G.S., Kappas A. (1987). Cobalt-prtoporphyrin suppresses thyroid and testicular hormone concentrations in rat serum: A novel action of this synthetic heme analogue. Pharmalogy.

[138] Bozza M.T., Jeney V. (2020). Pro-inflammatory actions of heme and other hemoglobin-derived DAMPS. Front. Immunol..

[139] Pamplona A., Ferreira A., Balla J., Jeney V., Balla G., Epiphanio S., Chora A., Rodrigues C.D., Gregoire I.P., Cunha-Rodrigues M. (2007). Heme oxygenase-1 and carbon monoxide suppress the pathogenesis of experimental malaria. Nat. Med..

[140] Larsen R., Gozzelino R., Jeney V., Tokaji L., Bozza F.A., Japiassu A.M., Bonaparte D., Marinho M., Cavalcante M.M., Chora A. (2010). A central role for free heme in the pathogenesis of severe sepsis. Sci. Transl. Med..

[141] Ogborne R.M., Rushworth S.A., Charalambos C.A., O’Connell M.A. (2004). Haem oxygenase-1: A target for dietary antioxidants. Biochem. Soc. Trans..

[142] Barbagallo I., Galvano F., Frigiola A., Cappello F., Riccioni G., Murabito P., D”orazio N., Torella M., Gazzolo D., Li Volti G. (2013). Potential therapeutic effects of natural heme oxygenase-1 inducers in cardiovascular disease. Antioxid. Redox Signal..

[143] Pergola P.E., Raskin P., Toto R.D., Meyer C.J., Huff J.W., Grossman E.B., Krauth M., Ruiz S., Audhya P., Christ-Schmidt H. (2011). Bardoxolone methyl and kidney function in CKD with type 2 diabetes. N. Eng. J. Med..

[144] De Zeeuw D., Akizawa T., Audhya P., Bakris G.L., Chin M., Christ-Schmidt H., Goldsberry A., Houser M., Krauth M., Heerspiink H.J.L. (2013). Bardoxolone methyl in type 2 diabetes and stage 4 chronic kidney disease. N. Eng. J. Med..

[145] Kanda H., Yamawaka K. (2020). Bardoxolone methyl: Drug development for diabetic kidney disease. Clin. Exp. Nephrol..

[146] Abdelrahman R.S., Abdel-Rahman N. (2019). Dimethylfumarate ameliorates acetaminophen-induced hepatic injury in mice dependent on Nrf2/HO-1 pathway. Life Sci..

[147] Behnammanesh G., Durante G.L., Khanna Y.P., Peyton K.J., Durante W. (2020). Canagliflozin inhibits vascular smooth muscle cell proliferation and migration: Role of heme oxygenase-1. Redox Biol..

[148] Neal B., Perkovic V., Mahaffey K.W., de Zeeuw D., Fulcher G., Erondu N., Shaw W., Law G., Desai M., Matthews D.R. (2017). Canagliflozin and cardiovascular and renal events in type 2 diabetes. N. Engl. J. Med..

[149] Liu X.M., Peyton K.J., Wang H., Durante W. (2012). Sildenafil stimulates the expression of gaseous monoxide-generating enzymes in vascular smooth muscle cells via distinct signaling mechanisms. Biochem. Pharmacol..

[150] Bharucha A.E., Choi K.M., Saw J., Gibbons S.J., Farrugia G., Carlson D., Zinsmeister A.R. (2014). Effects of aspirin & simvastatin, and aspirin, simvastatin & lipoic acid on heme oxygenase-1 in healthy human subjects. Neurogastroenterol. Motil..

[151] Mirjanic-Azaric B., Rizzo M., Jurgens G., Hallstroem S., Srdic S., Marc J., Cerne D. (2015). Atorvastatin treatment increases plasma bilirubin but not HMOX1 expression in stable angina patients. Scand. J. Clin. Lab. Investig..

[152] Hopper C.P., Meinel L., Stieger C., Otterbein L.E. (2018). Where is the clinical breakthrough of HO-1/carbon monoxide therapeutics. Curr. Pharm. Des..

[153] Wollborn J., Hermann C., Goebel U., Merget B., Wunder C., Maier S., Schafer T., Heuler D., Muller-Buschbaum K., Buerkle H. (2018). Overcoming safety challenges in CO-therapy–extracorporeal CO delivery under precise feedback control of systemic carboxyhemoglobin levels. J. Control Release.

[154] Foresti R., Motterlini R. (2010). Interaction of carbon monoxide with transition metals: Evolutionary insights into drug development. Curr. Drug Targets.

[155] Motterlini R., Foresti R. (2017). Biological signaling by carbon monoxide and carbon monoxide-releasing molecules. Am. J. Physiol. Cell Physiol..

[156] Nikam A., Ollivier A., Rivard M., Wilson J.L., Mebarki K., Martens T., Dubois J.L., Rande R., Motterlini R., Foresti R. (2016). Diverse Nrf2 activators coordinated to cobalt carbonyls induce heme oxygenase-1 and release carbon monoxide in vitro and in vivo. J. Med. Chem..

[157] Ali Z.E., Ollivier A., Manin S., Rivard M., Motterlini R., Foresti R. (2020). Therapeutic effects of CO-releaser/Nrf2 activator hybrids (HYCOs) in the treatment of skin wound, psoriasis and multiple sclerosis. Redox Biol..

[158] Wang D., Viennois E., Ji K., Damera K., Draganov A., Zheng Y., Dai C., Merlin D., Wang B. (2014). A click-and-release approach to CO prodrugs. Chem. Commun..

[159] Steiger C., Wollborn J., Gutmann M., Zehe M., Wunder C. (2015). Controlled therapeutic gas delivery systems for quality-improved transplants. Eur. J. Pharmcol. Biopharm..

[160] Belcher J.D., Gomperts E., Nguyen J., Chen C., Abdulla F., Kiser Z.M., Gallo D., Levy H., Otterbein L.E. (2018). Oral carbon monoxide therapy in murine sickle cell disease: Beneficial effects on vaso-occlusion, inflammation, and anemia. PLoS ONE.

[161] Schwertner H.A., Jackson W.G., Tolan G. (1994). Association of low serum concentration of bilirubin with increased risk of coronary artery disease. Clin. Chem..

[162] Mayer M. (2000). Association of serum bilirubin concentration with risk of coronary artery disease. Clin. Chem..

[163] Schwertner H.A., Vitek L. (2008). Gilbert syndrome, UGT1A1*28 allele, and cardiovascular disease risk: Possible protective effects and therapeutic applications of bilirubin. Atherosclerosis.

[164] Wang D.Q.-H., Carey M.C. (2014). Therapeutic uses of animal biles in traditional Chinese medicine: An ethnopharmacological, biophysical chemical and medicinal review. World J. Gastroenterol..

[165] Lee Y., Kim H., Kang S., Lee J., Park J., Jon S. (2016). Bilirubin nanoparticles as a nanomedicine for anti-inflammation. Angew. Chem. Int. Ed..

[166] Zhang D., Chando T., Everett D.W., Patten C.J., Dehal S.S., Humphreys G. (2005). In vitro inhibition of UDP glucuronosyltransferases by atazanavir and other HIV protease inhibitors and the relationship of this property to in vivo bilirubin glucuronidation. Drug Metab. Dispos..

[167] Liu X.-M., Durante Z.E., Peyton K.J., Durante W. (2016). Heme oxygenase-1-derived bilirubin counteracts HIV protease inhibitor-mediated endothelial cell dysfunction. Free Rad. Biol. Med..

[168] Dekker D., Dorresteijn M.J., Pijnenburg M., Heemskerk S., Rasing-Hoogveld A., Burger D.M., Wagener F.A.D.T.G., Smits P. (2011). The bilirubin-increasing drug atazanavir improves endothelial function in patients with type 2 diabetes mellitus. Arterioscler. Thromb. Vasc. Biol..

[169] Pattanawongsa A., Chau N., Rowland A., Miner J.O. (2015). Inhibition of human UDP-glucuronosyltransferase enzymes by canagliflozin and dapagliflozin: Implications for drug-drug interactions. Drug Metab. Dispos..

[170] Cefalu W.T., Leiter L.A., Yoon K.-H., Arias P., Niskanen L., Xie J., Balis D.A., Canovatchel W., Meininger G. (2013). Efficacy and safety of cangagliflozin versus glimepiride in patients with type 2 diabetes inadequately controlled with metformin (CANTATA-SU): 52 week results from a randomized, double-blind, phase 3 non-inferiority trial. Lancet.

[171] Lavalle-Gonzalez F.J., Januszewicz A., Davidson J., Tong C., Qiu R., Canovatchel W., Meininger G. (2013). Efficacy and safety of canagliflozin compared to placebo and sitagliptin in patients with type 2 diabetes on background metformin monotherapy: A randomized trial. Diabetologia.

